# Targeting CLDN18.2 by CD3 Bispecific and ADC Modalities for the Treatments of Gastric and Pancreatic Cancer

**DOI:** 10.1038/s41598-019-44874-0

**Published:** 2019-06-10

**Authors:** Guoyun Zhu, Davide Foletti, Xiaohui Liu, Sheng Ding, Jody Melton Witt, Adela Hasa-Moreno, Mathias Rickert, Charles Holz, Laura Aschenbrenner, Amy H. Yang, Eugenia Kraynov, Winston Evering, Leslie Obert, Chenyu Lee, Tao Sai, Tina Mistry, Kevin C. Lindquist, Thomas Van Blarcom, Pavel Strop, Javier Chaparro-Riggers, Shu-Hui Liu

**Affiliations:** 1 Pfizer Cancer Immunology Discovery, Pfizer Worldwide Research and Development, 230 E. Grand Avenue, South San Francisco, CA 94080 USA; 2 Drug Safety Research and Development, Pfizer Worldwide Research and Development, 10646 Science Center Dr., San Diego, CA 92121 USA; 3 BioMedicine Design, Pfizer Worldwide Research and Development, 10646 Science Center Dr., San Diego, CA 92121 USA; 4 Drug Safety Research and Development, Pfizer Worldwide Research and Development, 280 Shennecossett Rd, Groton, CT 06340 USA; 5 Present Address: 23 and Me, 349 Oyster Point Blvd, South San Francisco, CA 94080 USA; 6 Present Address: Gilead Sciences, 333 Lakeside Drive, Foster City, CA 94404 USA; 7 Present Address: Grifols Diagnostic Solutions, 6455 Christie Ave B-334C, Emeryville, CA 94608 USA; 8 Present Address: Kodiak Sciences Inc., 2631 Hanover St, Palo Alto, CA 94304 USA; 9 Present Address: Applied Molecular Transport, 1 Tower Place, Suite 850, South San Francisco, CA 94080 USA; 10 Present Address: Covance Inc. Early Phase Development Solutions, 3301 Kinsman Blvd, Madison, WI 53704 USA; 11 Present Address: GSK, 1250 South Collegeville Road, Collegeville, PA 19426 USA; 12 Present Address: Alector, 151 Oyster Point Blvd #300, South San Francisco, CA 94080 USA; 13 Present Address: Allogene Therapeutics, 210 E. Grand Avenue, South San Francisco, CA 94080 USA; 14 Present Address: Bristol-Myers Squibb, 700 Bay Rd suite A, Redwood City, CA 94063 USA; 15 Present Address: Multitude Therapeutics, Abmart, 3698 Haven Avenue Suite A, Redwood City, CA 94063 USA

**Keywords:** Cancer, Gastrointestinal cancer

## Abstract

Human CLDN18.2 is highly expressed in a significant proportion of gastric and pancreatic adenocarcinomas, while normal tissue expression is limited to the epithelium of the stomach. The restricted expression makes it a potential drug target for the treatment of gastric and pancreatic adenocarcinoma, as evidenced by efforts to target CLDN18.2 via naked antibody and CAR-T modalities. Herein we describe CLDN18.2-targeting via a CD3-bispecific and an antibody drug conjugate and the characterization of these potential therapeutic molecules in efficacy and preliminary toxicity studies. Anti-hCLDN18.2 ADC, CD3-bispecific and diabody, targeting a protein sequence conserved in rat, mouse and monkey, exhibited *in vitro* cytotoxicity in BxPC3/hCLDN18.2 (IC_50_ = 1.52, 2.03, and 0.86 nM) and KATO-III/hCLDN18.2 (IC_50_ = 1.60, 0.71, and 0.07 nM) respectively and inhibited tumor growth of pancreatic and gastric patient-derived xenograft tumors. In a rat exploratory toxicity study, the ADC was tolerated up to 10 mg/kg. In a preliminary assessment of tolerability, the anti-CLDN18.2 diabody (0.34 mg/kg) did not produce obvious signs of toxicity in the stomach of NSG mice 4 weeks after dosing. Taken together, our data indicate that targeting CLDN18.2 with an ADC or bispecific modality could be a valid therapeutic approach for the treatment of gastric and pancreatic cancer.

## Introduction

Gastric and pancreatic adenocarcinomas are diseases of malignant glandular cells. Approximately one million new cases of gastric cancer are diagnosed worldwide each year. Despite recent advances in treatment options, relapse is inevitable and patients become refractory to treatment. The five-year survival is about 5–20% and the median overall survival is about 10 months for patients with advanced gastric cancer^1–3^. For pancreatic cancer, the overall five-year survival rate is about 6~8%^3–5^. The poor prognosis of these two cancer types highlights the need for additional treatment approaches. One such approach is that of targeted therapies, an ever evolving field with promising modalities including antibody drug conjugates (ADCs) and CD3 bispecific antibodies now being tested in multiple indications^6–15^.

Covalently linking a cytotoxic “payload” to an antibody to form an ADC provides a mechanism for selective delivery of the cytotoxic agent to cancer cells via the specific binding of the antibody to cancer-selective cell surface molecules. Various payloads and linkers such as DNA damaging agents, microtubule inhibitors and cleavable or non-cleavable linkers can be combined to afford ADCs’ different characteristics^9,10^. FDA approvals of ado-trastuzumab emtansine (Kadcyla®; T-DM1) and inotuzumab ozogamicin (BESPONSA®) have validated this modality in the treatment of solid and hematological malignancies^16,17^. Despite these approvals, ADCs have limitations including undesired release of the toxic payload in circulation and off-target payload-related adverse events, such as lymphopenia/thrombocytopenia, which limit the maximum tolerated dose^18,19^. In addition, ADCs require a relatively high number of cell-surface tumor associated antigens (TAAs) per cell to achieve maximum efficacy^8,18^.

Cytotoxic T cells are considered to be the most potent effector cells of the immune system. In general, antigen-induced cytotoxic T-cell immunity is dependent on target cell antigen presentation and recognition of the presented peptide/MHC by the T-cell receptor (TCR), which includes the CD3 molecule. Bispecific antibodies can bind two different antigens simultaneously. By binding both a tumor target antigen and CD3, modalities such as CD3 bispecific antibodies can redirect T cells towards the recognition of tumor target antigen and induce T cell-mediated cell killing^20^. The idea of using bispecific antibodies to redirect circulating T cells to tumor sites *in vivo* and engaging them with cancer cells emerged in the 1980s^21,22^. The recent FDA approval of blinatumomab (the first-in-class bispecific T cell engager (BiTE) antibody against CD3–CD19) highlights the role of bispecifics as potentially transformative medicines^23,24^. T cell-redirecting bispecifics vary in format. This manuscript will focus on CD3 bispecific antibodies and diabodies.

Identifying a specific TAA for an oncology target that has limited normal tissue expression is critical. CLDN18.2 represents a potentially attractive TAA because it fulfills this criterion. The claudin multigene family encodes tetraspan membrane proteins that are crucial structural and functional components of tight junctions. In mammals, there are at least 27 claudin members identified and they exhibit complex tissue-specific patterns of expression. CLDN18.2 is highly expressed in the normal stomach and is strictly confined to differentiated epithelial cells of the gastric mucosa. Furthermore hCLDN18.2 is also expressed in a significant proportion of primary gastric cancers and their metastases, as well as in pancreatic and esophageal adenocarcinoma^25,26^. Recent studies have identified CLDN18-ARHGAP26/6 fusions in gastric cancers, with predominance in diffuse-type gastric cancers (DGCs). The patients with CLDN18-ARHGAP26/6 fusion have worse survival outcomes and show resistance to oxaliplatin/fluoropyrimidines-based chemotherapy^27,28^. Ganymed’s naked anti-CLDN18.2 antibody, Claudiximab, has been studied in numerous clinical trials for the treatment of patients with advanced gastroesophageal cancer. In combination with chemotherapy of epirubicin, oxaliplatin, and capecitabine (EOX), claudiximab showed promising results in gastric cancer patients in a phase II study by increasing progression free survival from 4.8 to 7.9 months and overall survival from 8.4 to 13.2 months^29,30^ relative to EOX chemotherapy regimen alone.

ADCs and bispecific antibodies represent promising therapeutic modalities for improving the clinical management of cancer. In order to test the efficacy of anti-hCLDN18.2 ADC and CD3-bispecific modalities, their cytotoxic activities were assessed *in vitro* against tumor cell lines engineered to express hCLDN18.2. In addition, patient-derived xenograft mouse models of pancreatic and gastric cancers were developed to assess the antitumor effects of the anti-CLDN18.2 ADC and CD3-bispecific *in vivo*. Overall, the anti-CLDN18.2 ADC and CD3-bispecific molecules were potent in killing tumor cells *in vitro* and inhibiting tumor growth in gastric and pancreatic cancer patient-derived xenograft (PDX) tumor models *in vivo*. Anti-hCLDN18.2 has similar cross-reactivity with mouse and monkey and the sequence of CLDN18.2 is 89~99% conserved between mouse, rat, monkey and human suggesting that both rat and mouse could be relevant species to assess toxicity. The ADC was tolerated up to 10 mg/kg in rats and the anti-CLDN18.2 diabody did not display obvious signs of toxicity in the stomach of tumor bearing NSG mice 4 weeks following a single dose of 0.34 mg/kg. Though the full nonclinical safety profile has yet to be elucidated, these data suggest that CLDN18.2 may be a promising therapeutic target for the treatment of gastric cancer and other CLDN18.2-expressing tumors. Our fındings offer preclinical proof-of-concept that the anti-CLDN18.2 ADC and anti-CLDN18.2-CD3 bispecific could be highly specific and potent against *in vivo* and *in vitro* models of gastric and pancreatic cancers.

## Results

### Expression profile of CLDN18.2 in the gastric and pancreatic cancer tissues

**Figure 1:**
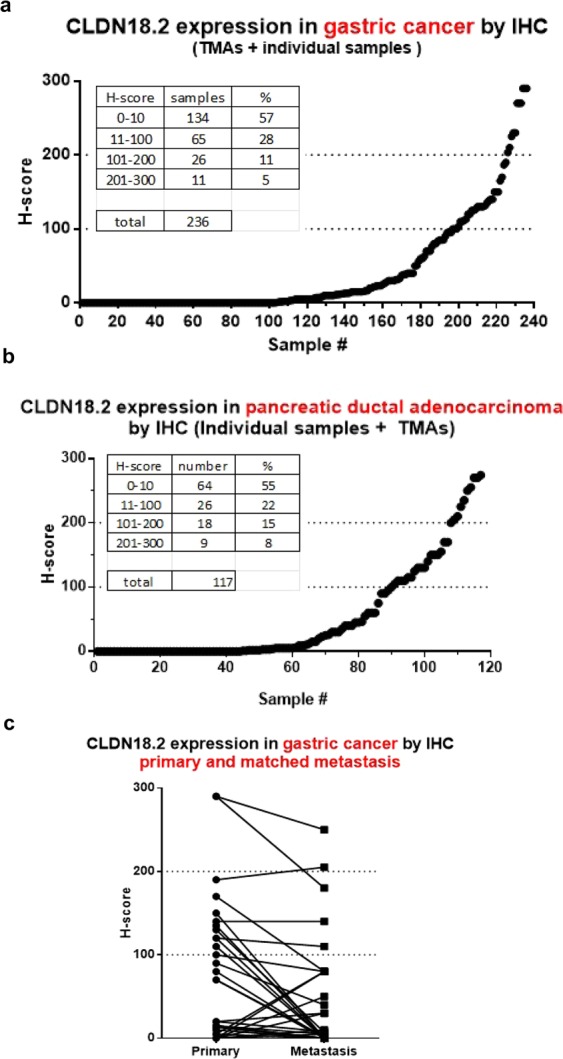
Gastric and pancreatic adenocarcinoma display CLDN18.2 expression (IHC). Tumor tissue samples of primary or metastatic gastric and primary pancreatic adenocarcinoma from tumor microarrays (Tristar or US Biomax) or individual samples were immunostained with an anti-hCLDN18.2 antibody, anti-CLDN-mlgG1, to determine the expression of CLDN18.2. Different levels of CLDN18.2 expression were evaluated by an experienced pathologist using a 4-point scale. H-score: plasma membrane staining intensity + distribution. Sum of staining intensity (0 = negative, 1 = low, 2 = medium, 3 = high) × percentage of cells. All grading is subjective and done by eye by a pathologist. The percentage of CLDN18.2-positive staining with different scores was indicated in the table embedded. (**a**) Primary gastric adenocarcinoma; (**b**) primary pancreatic adenocarcinoma; c. primary and metastatic matched gastric adenocarcinoma.

CLDN18.2 expression was assessed in 236 primary gastric cancer tissues and 117 pancreatic ductal adenocarcinoma tissues from tumor microarray (Tristar or US Biomax) or individual samples (Fig. 1a,b). 16–23% of gastric and pancreatic adenocarcinoma samples display mid to high (H score >100) CLDN18.2 expression. Additionally, CLDN18.2 was expressed in 34% (20/59) of the metastatic lesions (Fig. 1c). In general, the CLDN18.2 expression level in the metastatic lesions was relatively consistent with that in the corresponding primary gastric cancer tissues.

**Figure 2:**
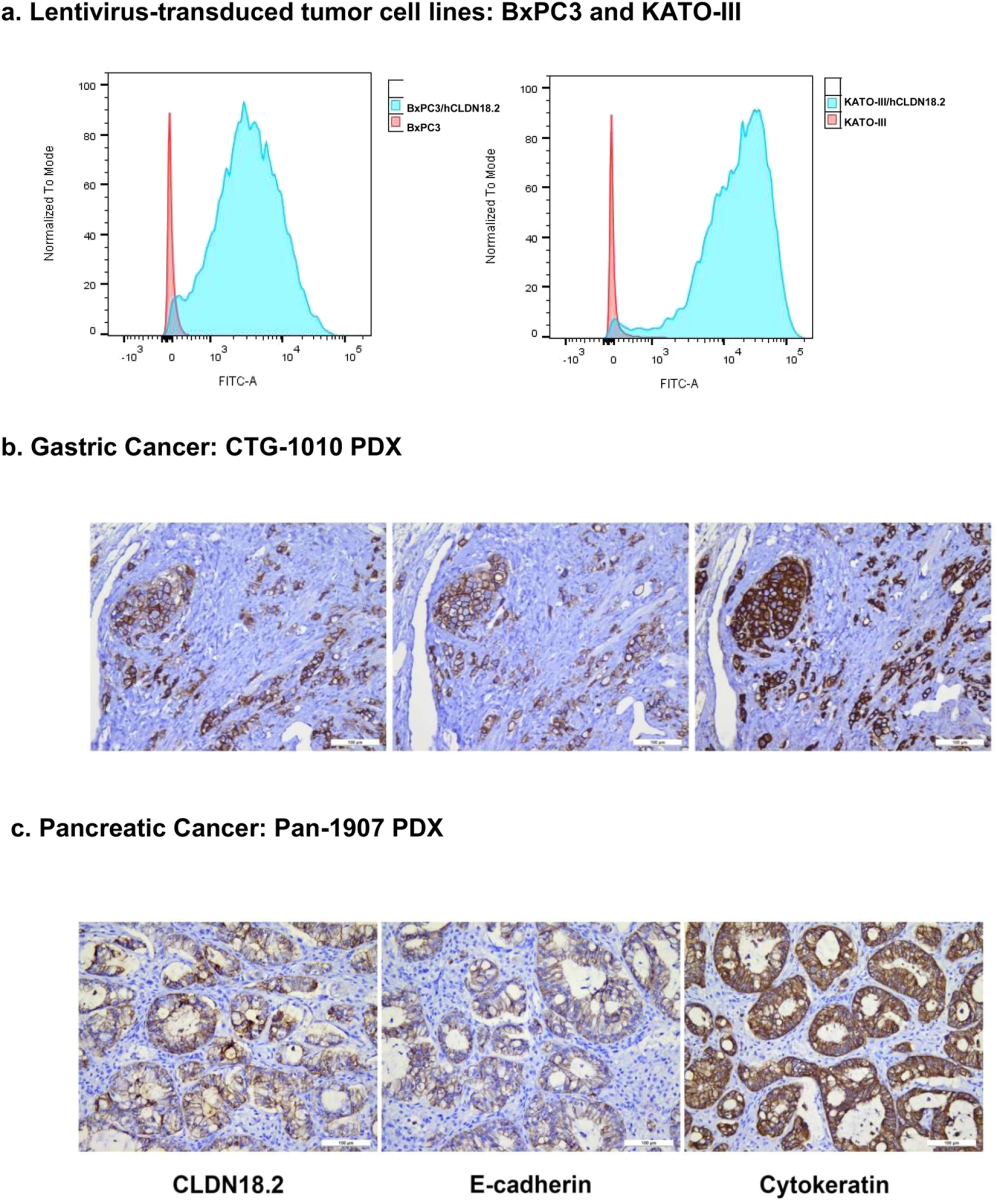
CLDN18.2 is expressed in Lentivirus-transduced tumor cell lines and gastric and pancreatic cancer PDX. Primary BxPC3, KATO-III and hCLDN18.2 engineered BxPC3, KATO-III cells were incubated with 10 μg/mL of anti-hCLDN18.2 mAb. Blue and red peaks represent staining with anti-CLDN18.2 in CLDN18.2 engineered expressing cells and non-engineered expressing cells respectively. (**a**) Patient-derived tumor sections, Gastric Cancer (CTG-1010, **b**) and Pancreatic Cancer (Pan-1907, **c**) were immunostained with an anti-CLDN18.2 antibody. Cytokeratin was stained with mouse anti-human cytokeratin mAb. E-Cadherin was stained with mouse anti-human E-Cadherin.

Since we did not identify uniform expression of CLDN18.2 in several gastric and pancreatic cancer cell lines tested, lentivirus transduced BxPC3/hCLDN18.2 and KATO-III/hCLDN18.2 cell lines were generated (Fig. 2a) for *in-vitro* characterization of the antibodies. Patient-derived gastric and pancreatic tumors that express hCLDN18.2 were also identified for *in vivo* characterization of the anti-CLDN18.2 ADC and CD3-bispecific molecules (Fig. 2b,c).

### Generation and characterization of anti-CLDN18.2 CD3 ADC, bispecific and diabody

An anti-hCLDN18.2 tool antibody was generated and was shown to be cross-reactive with cynomolgus monkey and mouse CLDN18.2 (data not shown). The anti-CLDN18.2 antibody was used as the backbone to prepare an ADC using site-specific transglutaminase conjugation^31^ with a valine-citrulline cleavable linker and an auristatin payload. The drug to antibody ratio is 4.

**Figure 3:**
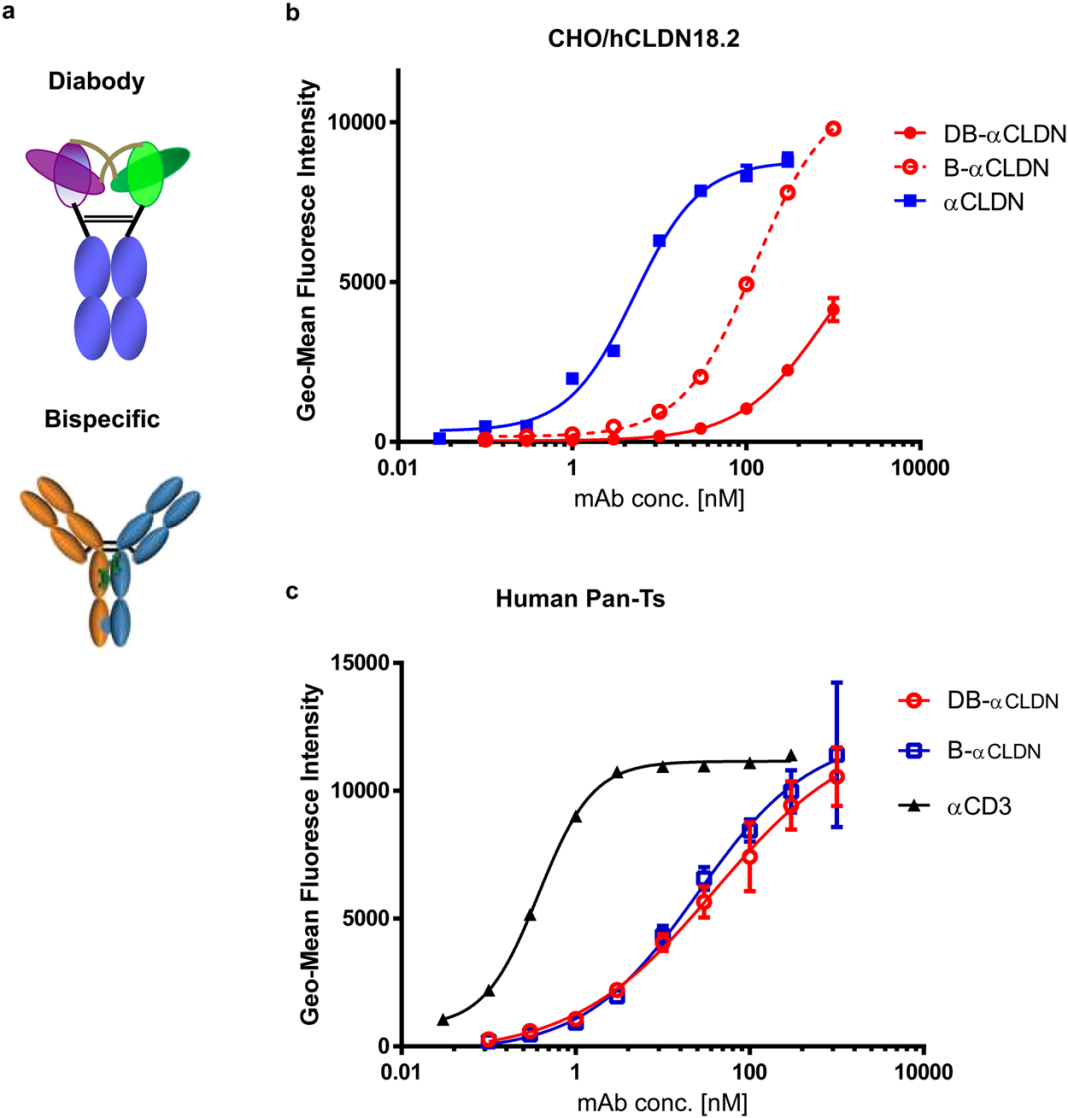
Anti-hCLDN18.2 or anti-hCD3 antibodies binds CHO/hCLDN18.2 or human Pan-T cells dose-dependently. Schematic of the two anti-CLDN18.2 therapeutic molecule formats: a full length fully human IgG2ΔA D265A bispecific or diabody generated by combining CLDN18.2 and CD3 targeting arms though hinge mutations. (**a**) CHO engineered expressing hCLDN18.2 and human Pan-T cells were assayed for anti-CLDN18.2-CD3 Bispecific and diabody cell binding. The anti-CLDN18.2 arm of the bispecific and diabody binds in a dose-dependent manner to CHO/CLDN18.2 cell line (**b**) and the anti-CD3 arm to Pan-T cells (**c**).

The tool antibody variable domains of anti-hCLDN18.2 were cloned into mammalian expression vectors for expression. A highly specific anti-CD3 antibody was generated in mice and humanized, resulting in an antibody binding to human and cynomolgus monkey CD3ε, respectively. The CLDN18.2 antibody was formatted into CD3 bispecific or diabody format using a hIgG2 Fc containing mutations that aid in heavy chain heterodimer formation^32^ and G2ΔA/D265A Fc mutations that reduce Fcγ receptor binding^33,34^ to prevent potential antibody clustering on immune cells and non-specific T-cell activation (Wei Chen and J. Chaparro-Riggers, manuscript in preparation). Schematics of both molecular formats are shown (Fig. 3a). The anti-CLDN18.2 antibody formatted into a CD3 bispecific or a diabody demonstrated effective binding to CLDN18.2-expressing CHO cell line or human T cells indicating that the bispecific format did not affect recognition of either epitope (Fig. 3b,c). While the binding of the bispecific antibody and the diabody is weaker than the parental antibody, this can be explained by avidity as the parental antibody is bivalent, while the bispecific antibody and diabody are monovalent.

### The anti-CLDN18.2 ADC and anti-CLDN18.2 CD3 bispecific molecules are effective mediators of tumor cell lysis *in vitro*

**Figure 4:**
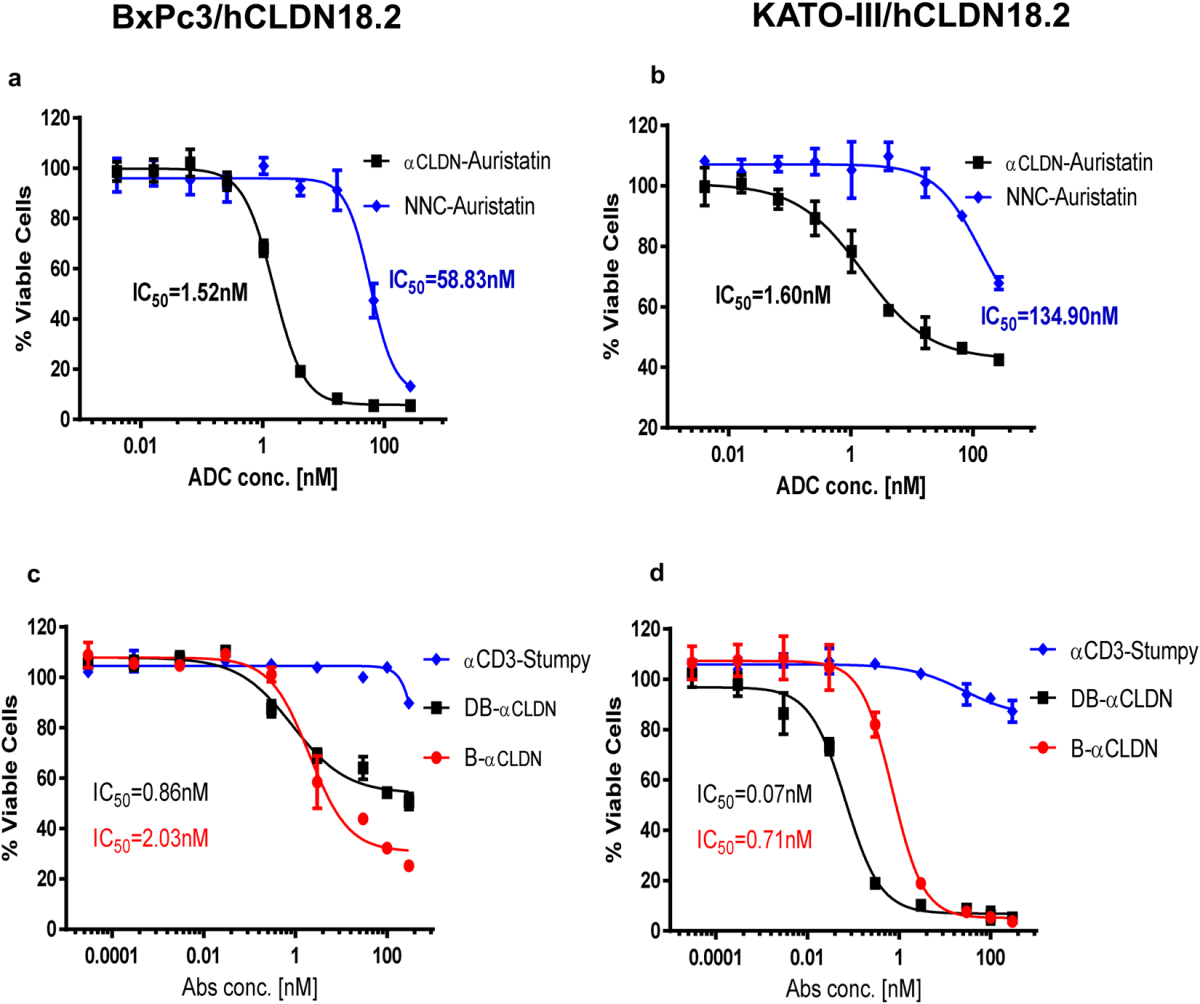
The anti-CLDN18.2 ADC and anti-CLDN18.2 CD3 Bispecific inhibit growth of different hCLDN18.2-tumor cells. The anti-CLDN18.2 ADC generated through site-specific conjugation of cleavable linkers and auristatin drug payloads with a drug-to-antibody ratio (DAR) of 4 dose-dependently inhibited the BxPc3 and KATO-III/hCLDN18.2 cells growth. (**a**,**b**) The target cells were incubated with varying concentrations of anti-CLDN18.2 cleavable ADC for 4 days. The cell viability was determined by luminescence by using CellTiter-Glo reagent (Promega). n = 3 technical replicates. NNC-Auristatin: anti-BHV-Auristatin. Bispecific and diabody also effectively eliminates BxPC3 or KATO-III/hCLDN18.2-Luc cells in a dose dependent manner. (**c**,**d**) Luciferase-labeled target cells were incubated with varying concentrations of the antibody and fixed ratio of Pan-T vs tumor cells (5:1) for 2 days. After 2 days of incubation, viability of BxPC3 or KATO-III cells were assessed using One-Glo luciferase assay reagent (Promega). The IC_50s_ were then determined by nonlinear regression plot of percent specific cytotoxicity versus Log10 concentration of CLDN18.2 bispecific using GraphPad Prism software, Version 7.04. The results were shown as mean ± standard deviation (SD). αCD3-stumpy: CD3 variable and constant region combined with a truncated antibody consisting of only the hinge/constant region.

In order to test the effect of the anti-CLDN18.2 ADC, the BxPc3 and KATO III cell lines stably engineered to express hCLN18.2 were used to determine the cytotoxic activity of the ADC. The anti-CLDN18.2 ADC exhibited *in vitro* cytotoxicity to BxPC3/hCLDN18.2 (IC50 = 1.52 nM) and KATO-III/hCLDN18.2 (IC50 = 1.60 nM) tumor cells (Fig. 4a,b).

To further evaluate the effect of the bispecific anti-CLDN18.2, the *in-vitro* cytotoxicity was tested in the same engineered tumor cell lines. The anti-CLDN18.2 bispecific and diabody mediated dose-dependent killing of hCLDN18.2-expressing tumor cells with IC_50_ values of 2.03 or 0.86 nM in BxPc3/hCLDN18.2 cells, respectively (Fig. 4c) and IC_50_ values of 0.71 or 0.07 nM in KATO III/hCLDN18.2 cells, respectively (Fig. 4d).

### The anti-CLDN18.2 ADC and anti-CLDN18.2 CD3 bispecific molecules exhibit efficacy against established gastric and pancreatic adenocarcinoma patient-derived tumors

**Figure 5:**
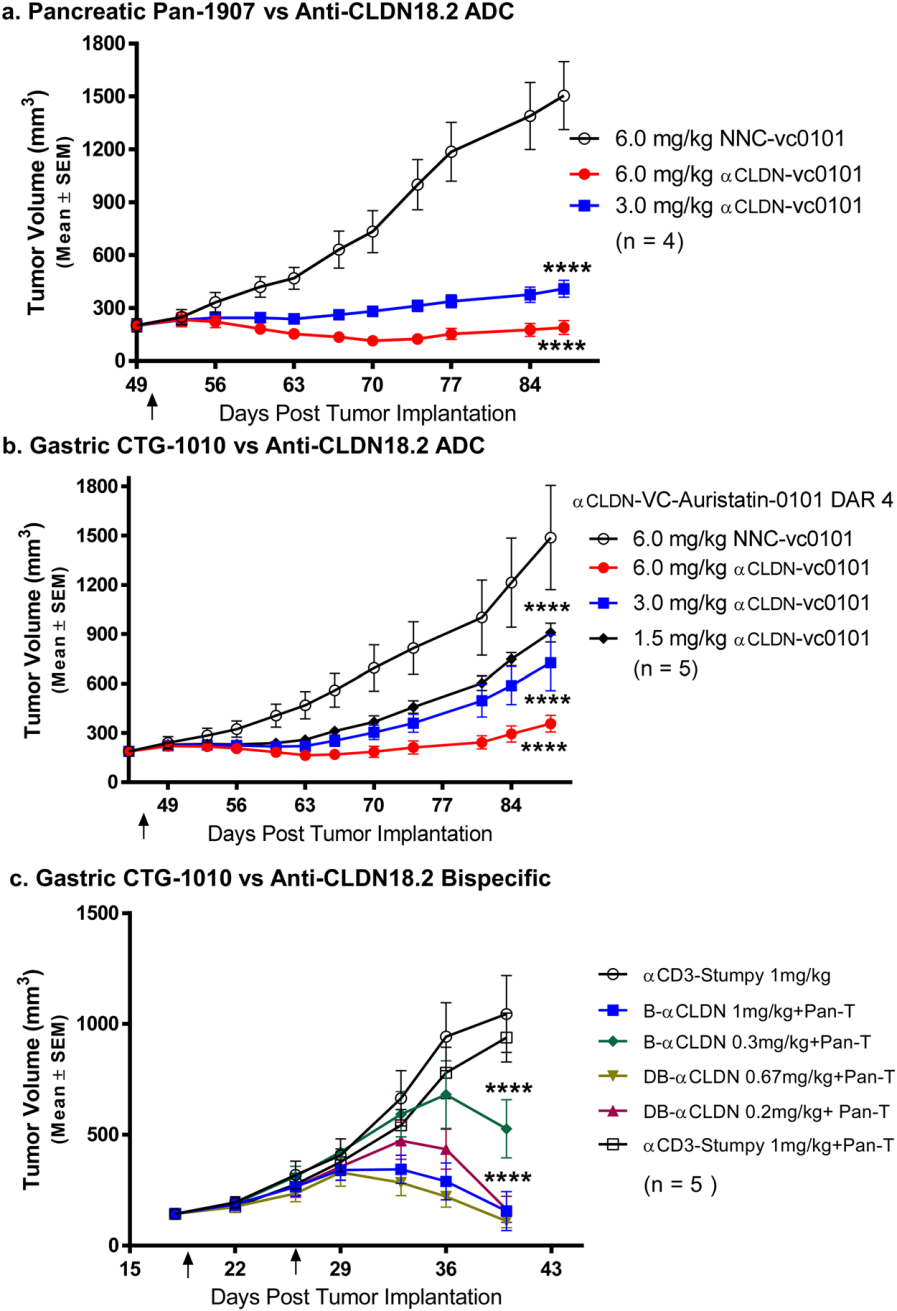
The anti-CLDN18.2 ADC and anti-CLDN18.2 CD3 Bispecific Inhibit tumor growths of Pancreatic Pan-1907 and Gastric CTG-1010 PDX. Anti-CLDN18.2 ADC with cleavable linker and DAR4 demonstrated dose-dependent efficacy against an established gastric and pancreatic patient-derived xenograft models after administering a single IV dose of Anti-CLDN18.2 ADC or Negative control (NNC) ADC 50 days (Pancreatic Pan-1907) or 46 days (Gastric CTG1010) after tumor inoculation. (**a**,**b**) Anti-CLDN18.2 CD3 bispecific and diabody were also efficacious at all doses tested in an established gastric patient-derived xenograft model. (**c**) All data shown as mean ± SEM. Tumor growth was monitored by caliper twice-weekly. All studies were performed in immune compromised NSG mice with n = 4–5 mice per group. Statistics represent two-way ANOVA analysis, all groups compared to NNC (for ADC, **a**,**b**) or αCD3-Stumpy (for bispecific, **c**) ****p < 0.0001).

Patient-derived xenograft models were chosen for *in vivo* studies. In these models, fresh pancreatic adenocarcinoma tumor blocks (Pan-1907 PDX) or gastric adenocarcinoma (CTG-1010) were resected from mice, cut into 2 mm^3^ cubes, and subcutaneously implanted into the right flanks of female NSG mice. After tumor establishment, animals were randomized based on tumor volume and a single dose of the anti-CLDN18.2 ADC was administered intravenously. Tumor growth inhibitory activity was seen at all ADC doses tested following a single injection and appeared to be dose-dependent in the CLDN18.2-expressing gastric and pancreatic PDX tumor models (Fig. 5a,b).

The same gastric PDX model was also used to assess *in vivo* efficacy of the anti-CLDN18.2 CD3 bispecific and diabody molecules. After tumor establishment, animals were randomized based on tumor volume and administered 2 × 10^7^ expanded human T cells by intraperitoneal (IP) injection. One and eight days later, animals were administered the CLDN18.2 bispecific or diabody molecules or the negative control CD3-Stumpy (CD3 variable and constant region combined with a truncated antibody consisting of only the hinge/constant region). Tumor regression was seen in a dose-dependent manner with diabody showing better *in vivo* efficacy (Fig. 5c).

### Characterization of the anti-CLDN18.2 ADC toxicity in a rat exploratory toxicity study and anti-CLDN18.2 diabody in tumor bearing NSG mice

**Figure 6:**
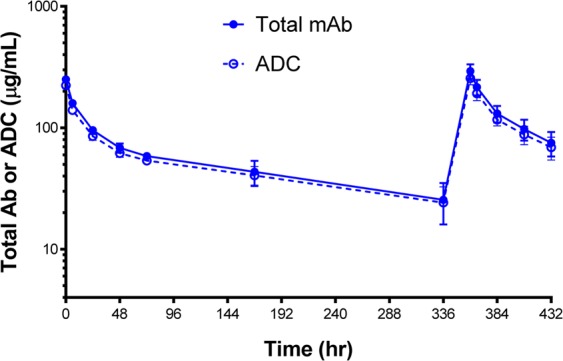
Anti-CLDN18.2 ADC serum concentration-time profiles. Anti-CLDN18.2 ADC serum concentration-time profiles (total mAb and ADC) in rats after intravenous administration of 10 mg/kg on days 1 and 15. Concentration of total mAb and ADC, determined using an ELISA, are shown as solid and dashed lines, respectively.

Anti-hCLDN18.2 has similar cross-reactivity with mouse and the sequence of CLDN18.2 is 89~99% conserved between mouse, rat, monkey and human suggesting that both rat and mouse could be relevant species to assess toxicity. An exploratory rat study was performed to evaluate the toxicity and pharmacokinetic (PK) of the anti-CLDN18.2 ADC modality. The compound exhibited PK (ADC half-life of 10 ± 3 days) consistent with that typically observed with ADCs. No loss of the cytotoxic payload was observed in systemic circulation based on the similarity of PK profiles for total antibody and intact ADC (Fig. 6).

Animals administered 25 mg/kg were moribund on Day 3 and euthanized. However, the anti-CLDN18.2 ADC was tolerated up to 10 mg/kg for 18 days with no ADC-related clinical signs of toxicity observed. Due to the known expression of CLDN18.2, microscopic findings such as mild to moderate increased mitoses/single cell necrosis in the glandular stomach were likely related to the killing of CLDN18.2-expressing cells. Other findings included decreased total white blood cells and neutropenia, decreased red blood cell parameters, which were correlated with microscopic changes of moderate to marked decreased cellularity of the bone marrow and lymphoid tissues. In addition, at 10 mg/kg ADC, microscopic analysis revealed minimal edema of the lung and minimal to mild increased mitoses/single cell necrosis in the eyes (corneal epithelium), liver (hepatocytes, sinusoidal cells, and bile ducts), heart (interstitial cells), kidneys (interstitial cells) and intestines (epithelial cells of cecum and colon) and higher AST (1.5x) and GLDH (2.4x), relative to controls. These findings were likely findings related to ADC payload toxicity since there is no known expression of CLDN18.2 in these tissues^26,35^.

Given the localized CLDN18.2 expression in the normal stomach, a histopathologic evaluation was performed on the stomach of tumor-bearing NSG female mice 4 weeks after a single intravenous dose (0.34 mg/kg) of an anti-CLDN18.2 diabody. There were no anti-CLDN18.2 diabody-associated stomach findings.

## Discussion

CLDN18.2, a splice variant of claudin 18, is highly expressed in normal stomach tissue and is strictly confined to differentiated epithelial cells of the gastric mucosa^26^. hCLDN18.2 is also expressed in a significant proportion of primary gastric cancers and their metastases, pancreatic and esophageal adenocarcinomas. Because of the restricted expression of CLDN18.2, it could be a potential target for various platforms of targeted therapies. To further confirm the presence of CLDN18.2 in different tumor tissues, we assessed its expression in primary gastric cancer and pancreatic tissues as well as metastatic gastric lesions by IHC. The results demonstrated that expression levels of CLDN18.2 in the metastatic gastric lesions and their primary cancer tissues were relatively consistent with each other. However, the percentage of tumors (16–23%) with higher CLDN18.2 expression in gastric and pancreatic tumors assessed in this study is much lower than what has been previously reported (60~73%)^26,36^. Possible reasons to explain this discrepancy include the sampling of tissues collected from different populations, staining conditions and sample quality. We did notice that different batches of tumor microarray samples from two different vendors had significantly different CLDN18.2 expression (65% vs 31% CLDN18.2 positive).

In the current study, the CLDN18.2 ADC successfully lysed gastric and pancreatic adenocarcinoma cells with varying levels of CLDN18.2 cell-surface expression. The data showed that BxPC3 cell line is more sensitive to ADC than KATO-III although the IC_50_ values are similar. However, the bispecific and diabody did display better cell killing of the gastric KATO-III/CLDN18.2 cells than the pancreatic cancer cell line, BxPC3/hCLDN18.2. The reasons for these differences remain to be elucidated but they are unlikely to be related to target density as the KATO-III/CLDN18.2 cells have higher engineered expression of the target compared to BxPC3/CLDN18.2 cells. Moreover, mechanisms of cell killing are different between ADC and bispecific.

To evaluate CLDN18.2-CD3 bispecific and CLDN18.2 ADC efficacy *in vivo*, we developed PDX models, utilizing patient-derived tumor blocks and activated human T cells in immune-compromised NSG mice. The PDX model approach allows for direct engraftment of human tumor cells and preserves tumor heterogeneity and lineage hierarchy^37^. Tumor blocks were implanted subcutaneously into the immune-compromised NSG mice. Both modalities were efficacious, but the bispecific, especially the diabody modality, was more potent compared to the ADC.

A rat exploratory toxicology study was performed to evaluate the toxicity of the ADC and assess its PK. The intact ADC and total antibody systemic PK profiles were similar throughout the entire time course, suggesting overall stability of the ADC in circulation in that the payload remained linked to the antibody until it reached its target destination and was internalized by cells. Administration of the ADC was tolerated up to 10 mg/kg in rat. Toxicity consistent with CLDN18.2 expression was seen with the ADC molecule and included increased mitoses/single cell necrosis in the stomach. In general, additional findings in other tissues were consistent with toxicity of auristatin payload and could be related to either small amounts of free payload in systemic circulation or non-specific distribution of the ADC to other tissues as there is no significant CLDN18.2 expression in these tissues^25,26,35^. Although we did not perform a rat exploratory toxicology study with the bispecific antibody, histopathological analysis in the stomach of gastric cancer PDX bearing NSG mice 4 weeks after dosing with the diabody did not show diabody-associated findings. These observations are likely an underestimate of toxicity or efficacy: since the histopathologic evaluation was performed 4 weeks after a single administration of Pan-T cells and the diabody, any potential damage in the stomach may have already had the chance to recover due to the rapid turnover of gastric epithelial cells^38^. Notably, Jiang and colleagues reported that administration of an mouse cross reactive anti-CLDN18.2 CAR T to mice (using hu8E5–2I as the antigen-binding element) exhibited no obvious toxicities in the stomach^36^. Similarly, preliminary results from a phase I clinical trial with Claudin18.2-CAR-T cells (treatment of pancreatic and gastric cancer with an enrollment of 12 patients) showed that the therapy was well-tolerated. After optimization of the dosing regimen, 5 patients from a 6-subject sub-cohort had objective response^39^. Although the binding affinity of the antibody or the expression level of the antigen and the different platforms may affect the on-target off-tumor or off-target cytotoxicities, bispecific modalities may represent another feasible clinical approach to treat gastric and pancreatic cancer patients.

In summary, while both modalities appear promising and potentially warrant further preclinical development, nonclinical efficacy data suggest the CD3 bispecific targeting hCLDN18.2 would have the potential to be a valuable therapeutic in the clinical setting.

## Materials and Methods

### Antibody expression and purification

The tool antibody variable domains of anti-hCLDN18.2 antibody were synthesized at GeneArt or ATUM and cloned into mammalian expression vectors for full length hIgG2dA D265A that carry 223E, 225E, 228E, and 368E mutations. The anti-CD3 arm was cloned into hIgG2dA D265A vectors that carry 223R, 225R, 228R, and 409R mutations. Parental antibodies were expressed separately using Expi293 system (Thermo Fisher), purified by MabSelect SuRe (GE), and then buffer exchanged into PBS. hIgG2dA D265A heterodimers were then prepared in a manner similar to as previously described^32^. Briefly, CLDN18.2 and CD3 parental antibodies were mixed with equal molar ratio, with addition of 1 mM reduced glutathione at 37 °C overnight, followed by re-oxidation with 1 mM oxidized glutathione at 37 °C. The desired bispecific antibodies were purified and separated from remaining parental antibodies using MonoS (GE) columns on an Akta Avant (GE). Protein was loaded in Buffer A (25 mM MES, pH 5.5, 20 mM NaCl) and eluted with a linear gradient of 0–50% of Buffer B (25 mM MES, pH 5.5, 1 M NaCl) over 40 column volumes.

To make the bispecific diabodies, anti-hCLDN18.2 VH or VL fragments were ligated separately to VL or VH of anti-CD3, respectively, with a short GS linker in between (GGGSGGGG). The variable domain fragments were fused to hIgG2dA D265A Fc with mutation pairs similar to what was previously described^32^, which favored the desired heterodimer formation. The resulting vectors for anti-CLDN_VL-GS linker-anti-CD3_VH-Fc and anti-CD3_VL-GS linker-anti-CLDN_VH-Fc were then co-expressed (with 1:1 DNA molar ratio) in Expi293 system (Thermo Fisher). The desired diabody products were purified by MabSelect SuRe followed by MonoS.

Final products of bispecific antibody and diabody were formulated in PBS for further studies.

Antibody drug conjugates were generated based on methods previously described^31^. Anti-CLDN18.2 antibody was conjugated to the cleavable auristatin, AcLysValCitPABC-Auristatin-0101 as described^40^. Briefly, antibody concentration was adjusted to 5 mg/mL in buffer containing 25 mM Tris-HCl, pH 7.5–8.0, 150 mM sodium chloride. Linker-payload was added in a 10- to 30-fold molar excess over antibody, and the enzymatic reaction was initiated by addition of 2% (w/v) bacterial transglutaminase (Ajinomoto Activa TI, Japan). Following incubation with shaking at 37 °C for 16 h, conjugates were purified using preparative Butyl Sepharose High Performance (Butyl HP, GE Healthcare Biosciences). Fractions containing the ADC were pooled, dialyzed against PBS, concentrated using a 10 kDa Amicon Ultra centrifugal filter unit (Millipore Corporation), and sterile filtered through a 0.2 μm filter.

### Cell culture

Gastric and pancreatic cancer cell lines (BxPc3 and KATO III) and CHO were obtained from ATCC. All cells were cultured according to the supplier’s recommendations and were maintained in a humidified chamber at 37 °C, 5% CO_2_. Cell lines were engineered to express hCLDN18.2 or hCDLN18.2-Luciferase using pLVX-EF1a-Puro-P2A or pLVX-EF1a-Luc2-P2A lentivirus (GenScript). Peripheral blood mononuclear cells (PBMC) were sourced from Stanford blood center, Palo Alto, CA and Pan-T cells were isolated using human Pan T Isolation Kit (Miltenyi Biotec).

### *In vitro* cytotoxicity assays

Bispecific cytotoxicity assays were performed in a 96-well plate format by mixing purified human CD3^+^ T cells and luciferase-labeled BxPC3/hCLDN18.2 or KATO-III/hCLDN18.2 cells, with an effector to target ratio (E:T) of 5:1. Serial dilutions of bispecific antibody were added to the plates and after 2 days of incubation, cell viability was assessed with the One-Glo luciferase reagent (Promega).

ADC assays were performed by plating BxPC3/hCLDN18.2 or KATO-III/hCLDN18.2 cells and adding serial dilutions of ADCs. After 4 days of incubation, viability of the cells was assessed by using CellTiter-Glo reagent (Promega).

### T-cell activation and expansion

Pan-T cells isolated from blood samples obtained from the Stanford Blood Center were thawed and activated by adding human T-cell activation and expansion MACSi Beads at 1:2 bead-to-cell ratios for 72 hours. On day 3 after activation, cells were transferred to a Grex-100 flask (Wilson Wolf). The interleukin (IL)-2 (Miltenyi Biotec) was added at a final concentration of 100 units/mL. Additional IL-2 was added every three days. Media (300 mL) was removed and replaced on Day 7 after activation. On Day 11, T cells were spun at 1500 RPM for 10 minutes and resuspended in culture media. Activation beads were removed with a magnet, and viability and the number of T cells was determined. Cells were spun again and resuspended in cold PBS for injection.

### Patient-derived xenograft (PDX) tumor models

Animal studies (mice) were carried out under protocols approved by the Pfizer Animal Care and Use Committee and performed in an AAALAC accredited facility. All methods were performed in accordance with the relevant guidelines and regulations. Fresh pancreatic adenocarcinoma tumor blocks (Pan-1907 PDX) or gastric adenocarcinoma tumor blocks (CTG-1010) resected from mice were cut into cubes of 2 mm^3^ and were subcutaneously implanted into the right flanks of 6-week-old female NSG mice (The Jackson Laboratory). After tumor establishment, animals were randomized based on tumor volume and administered 2 × 10^7^ expanded human T cells by IP injection for bispecific and diabody studies. One day later, animals were intravenously administered the first dose of bispecific or CD3-stumpy (CD3 variable and constant region combined with a truncated antibody consisting of only the hinge/constant region, thus a single arm with no tumor antigen targeting). A second dose of bispecific or CD3-stumpy was administered to CTG-1010 PDX repeat-dose groups 1 week following the initial dose. Tumor volume was measured twice a week with a caliper device and calculated with the following formula: Tumor volume = (length × width^2^)/2. Animals were euthanized once their tumor volumes reached 2000 mm^3^. ADC studies were performed similarly, without exogenous T cells. The ADC was injected on Day 46 for gastric CTG-1010 PDX or on Day 50 for pancreatic Pan-1907 PDX. NNC for ADC was anti-BHV.

### Rat study: Toxicity-pharmacokinetic analyses

An exploratory rat study was performed to evaluate the toxicity and pharmacokinetic (PK) parameters of the anti-CLDN18.2 ADC modality. The study protocol for the rat toxicology work was approved by the Animal Care and Use Committee of the AAALAC accredited institution (Pfizer Inc) and all methods were performed in accordance with the relevant guidelines and regulations. The ADC (10 or 25 mg/kg) and vehicle control (PBS) was administered to 3 male Wistar rats per group on Days 1 and 15. Blood samples were collected throughout the study for pharmacokinetic analysis and clinical pathology assessments. Subsequently on Day 18, tissues were collected from surviving animals for histopathological evaluation by a board certified veterinary pathologist.

Serum samples were analyzed using an ELISA developed on GyroLab immunoassay platform. For the total mAb, CLDN18.2 ADC was captured using biotinylated polyclonal goat anti-human IgG (H + L) antibody (Southern Biotech) and detected with a polyclonal goat anti-human IgG (H + L) (Bethyl Laboratories) labeled with Alexa Fluor 647. For the ADC assay, CLDN18.2 ADC was captured with biotinylated polyclonal goat anti-human IgG (H + L) antibody (Southern Biotech). The detection of the captured CLDN18.2 ADC was done with a polyclonal antibody generated in-house that recognizes the payload. The instrument response was used to construct a standard curve and calculate concentration of study samples and QCs. For both antibody and ADC assays, the lower limit of quantitation was 50 ng/mL. Pharmacokinetic data analysis was performed by the noncompartmental method using Phoenix software v. 6.3 (Pharsight).

#### Hematology and clinical chemistry

Blood samples were collected from surviving animals on Days 8, 15 and 18 for standard hematology and clinical chemistry panels.

#### Microscopic analysis

Animals were euthanized on Day 18 for necropsy. During necropsy, tissues were examined for gross lesions and a panel of tissues (large and small intestine, eye, liver, lung, pancreas, kidney, stomach) was collected, preserved in10% neutral buffered formalin (except for eye in 3% glutaraldehyde). Tissues were sectioned, processed to slides and stained with hematoxylin and eosin for microscopic analysis.

### Preliminary assessment of tolerability in NSG mice

CTG-1010 gastric cancer PDX bearing NSG mice were dosed once with anti-CLDN18.2 diabody (administered at 0.34 mg/kg, IV) and activated Pan-T cells (20 million cells, IP). The stomach was collected 4 weeks after dosing for histopathological analysis. All slides were assessed by a board certified veterinary pathologist.

### Immunohistochemistry and scoring

Formalin-fixed, paraffin-embedded tumor sections or tumor microarrays were processed and stained with anti-CLDN18.2 mAb, followed by detection with EnVision HRP-labeled polymer anti-rabbit second Ab (Dako K4002) based on standard protocol.

#### Scoring

Semi-quantitative assessments of the immunohistochemical stain results were performed by a pathologist. Only membranous (partial or complete) staining was defined as positive. The staining intensity of tumor cells was graded as weak (1+), moderate (2+) or strong (3+). An IHC H score was calculated by multiplying staining intensity (1+ to 3+) by the percentage of positive cells (0–100%) for each intensity for a final IHC H score of 0–300^41,42^.

### Statistical analysis

Statistical analysis was performed using the Prism software package (GraphPad).

## Data Availability

All data and associated experimental methods are displayed in the manuscript.
