# Author Correction: Targeting CLDN18.2 by CD3 Bispecific and ADC Modalities for the Treatments of Gastric and Pancreatic Cancer

**DOI:** 10.1038/s41598-019-53130-4

**Published:** 2019-11-08

**Authors:** Guoyun Zhu, Davide Foletti, Xiaohui Liu, Sheng Ding, Jody Melton Witt, Adela Hasa-Moreno, Mathias Rickert, Charles Holz, Laura Aschenbrenner, Amy H. Yang, Eugenia Kraynov, Winston Evering, Leslie Obert, Chenyu Lee, Tao Sai, Tina Mistry, Kevin C. Lindquist, Thomas Van Blarcom, Pavel Strop, Javier Chaparro-Riggers, Shu-Hui Liu

**Affiliations:** 1 Pfizer Cancer Immunology Discovery, Pfizer Worldwide Research and Development, 230 E. Grand Avenue, South San Francisco, CA 94080 USA; 2 Drug Safety Research and Development, Pfizer Worldwide Research and Development, 10646 Science Center Dr., San Diego, CA 92121 USA; 3 BioMedicine Design, Pfizer Worldwide Research and Development, 10646 Science Center Dr., San Diego, CA 92121 USA; 4 Drug Safety Research and Development, Pfizer Worldwide Research and Development, 280 Shennecossett Rd, Groton, CT 06340 USA; 5 Present Address: 23 and Me, 349 Oyster Point Blvd, South San Francisco, CA 94080 USA; 6 Present Address: Gilead Sciences, 333 Lakeside Drive, Foster City, CA 94404 USA; 7 Present Address: Grifols Diagnostic Solutions, 6455 Christie Ave B-334C, Emeryville, CA 94608 USA; 8 Present Address: Kodiak Sciences Inc., 2631 Hanover St, Palo Alto, CA 94304 USA; 9 Present Address: Applied Molecular Transport, 1 Tower Place, Suite 850, South San Francisco, CA 94080 USA; 10 Present Address: Covance Inc. Early Phase Development Solutions, 3301 Kinsman Blvd, Madison, WI 53704 USA; 11 Present Address: GSK, 1250 South Collegeville Road, Collegeville, PA 19426 USA; 12 Present Address: Alector, 151 Oyster Point Blvd #300, South San Francisco, CA 94080 USA; 13 Present Address: Allogene Therapeutics, 210 E. Grand Avenue, South San Francisco, CA 94080 USA; 14 Present Address: Bristol-Myers Squibb, 700 Bay Rd suite A, Redwood City, CA 94063 USA; 15 Present Address: Multitude Therapeutics, Abmart, 3698 Haven Avenue Suite A, Redwood City, CA 94063 USA

Correction to: *Scientific Reports* 10.1038/s41598-019-44874-0, published online 10 June 2019

This Article contains a typographical error in the Materials and Methods section under subheading ‘Immunohistochemistry and scoring’ where,

“Formalin-fixed, paraffin-embedded tumor sections or tumor microarrays were processed and stained with anti-CLDN18.2 mAb, followed by detection with EnVision HRP-labeled polymer anti-rabbit second Ab (Dako K4002) based on standard protocol.”

should read:

“Formalin-fixed, paraffin-embedded tumor sections or tumor microarrays were processed and stained with anti-CLDN18.2 mAb, followed by detection with EnVision HRP-labeled polymer anti-mouse second Ab (Dako K4001) based on standard protocol.”

